# A Rapid Differential Method for the Isolation of Bacillus Influenzae

**Published:** 1919

**Authors:** 


***A Rapid Differential Method for the Isolation of Bacillus Influenzae.*** By James Howard Brown and Marion L. Orcutt. *The Journal of Experimental Medicine*, Nov. 1, 1918.

Blood agar plates were seeded uniformly with a pure culture of Bacillus influenzae and were then streaked with hemolytic and non-hemolytic staphylococci, hemolytic and non-hemolytic streptococci, hemolytic and non-hemolytic strains of Micrococcus catarrhalis. After incubation it was found that the more luxuriant growth of Bacillus influenzae occurred only in the neighborhood of the streaks of hemolytic staphylococci, streptococci, and micrococci, not in the neighborhood of the non-hemolytic strains.

The more luxuriant growth of Bacillus influenzae in the neighborhood of colonies of hemolytic cocci is the result of the presence of released hemoglobin diffusing outward from those colonies.

This phenomenon has been utilized for the isolation of Bacillus influenzae from sputum. After blood agar plates have been inoculated with sputum, each of them is streaked with a pure culture of markedly hemolytic staphylococcus or streptococcus. If colonies of Bacillus influenzae are present, they will be found in largest number and of largest size in the vicinity of the zone of hemolysis produced by the streak of staphylococcus or streptococcus.

